# Differential Requirement for Cathepsin D for Processing of the Full Length and C-Terminal Fragment of the Malaria Antigen MSP1

**DOI:** 10.1371/journal.pone.0024886

**Published:** 2011-10-28

**Authors:** Calogero Tulone, Anne-Marit Sponaas, Eun-Ang Raiber, Alethea B. Tabor, Jean Langhorne, Benny M. Chain

**Affiliations:** 1 Division of Infection and Immunity, University College London, London, United Kingdom; 2 Division of Parasitology MRC National Institute of Medical Research, London, United Kingdom; 3 Department of Chemistry, University College London, London, United Kingdom; Tulane University, United States of America

## Abstract

Merozoite Surface Protein 1 is expressed on the surface of malaria merozoites and is important for invasion of the malaria parasite into erythrocytes. MSP1-specific CD4 T cell responses and antibody can confer protective immunity in experimental models of malaria. In this study we explore the contributions of cathepsins D and E, two aspartic proteinases previously implicated in antigen processing, to generating MSP1 CD4 T-cell epitopes for presentation. The absence of cathepsin D, a late endosome/lysosomal enzyme, is associated with a reduced presentation of MSP1 both following in vitro processing of the epitope MSP1 from infected erythrocytes by bone marrow-derived dendritic cells, and following *in vivo* processing by splenic CD11c+ dendritic cells. By contrast, processing and presentation of the soluble recombinant protein fragment of MSP1 is unaffected by the absence of cathepsin D, but is inhibited when both cathepsin D and E are absent. The role of different proteinases in generating the CD4 T cell repertoire, therefore, depends on the context in which an antigen is introduced to the immune system.

## Introduction

Protective immunity against blood-stage malaria is dependent on CD4 T cells and B cells [1]. In *Plasmodium chabaudi* (AS) infections in mice, development of IFNγ-producing Th1 cells and antibody are required to control parasitemia. CD4 Th1 T cells are also implicated in driving inflammatory responses linked to pathology. Despite the importance of CD4 T cells in malaria infections, the factors governing their activation, differentiation and regulation are not fully understood.

The primary activation of CD4 T cells requires that the antigen is processed and presented by dendritic cells (DC) [2]. Protein antigens are taken up by endocytosis or phagocytosis, and cleaved by intracellular proteinases to yield short peptides 10–20 amino acids in length. The peptides bind to class II Major Histocompatibility Complex (MHC) within endosomes, and the peptide/MHC complex is then displayed at the cell surface for subsequent recognition by specific T cells. The interaction between a specific T cell receptor and its cognate MHC/peptide complex is the primary recognition driving subsequent T cell activation, differentiation and proliferation.

Despite the central importance of antigen processing in the overall CD4 T response, many of the details of which enzymes are required, their specificity, and their role in shaping the repertoire of the response, remain unclear. Understanding these intracellular events is nevertheless important, since the repertoire of peptides displayed can influence the response both quantitatively and qualitatively.

MSP1 is expressed as a 200 kD protein on the surface of merozoites [3]. All but the C-terminal 19 kD fragment (MSP1_19_) is cleaved shortly before the merozoite invades the RBC [4]. MSP1_19_ is considered as a malaria vaccine candidate and in rodents, high levels of specific antibody can confer protection [4]. In natural human infections, however, MSP1_19_-specific antibody responses can be short-lived and comparatively low, despite repeated exposure to infection [5].

The tightly folded structure of MSP1_19_ is stabilized by five or six disulfide bonds which can limit antigen processing and, thereby, may affect the generation of CD4+ T cells providing help for B cells [6]. In *Plasmodium chabaudi*, the equivalent to the *P.falciparum* C-terminal MSP1_19_, PcMSP1_21_, contains some CD4 T cell epitopes which requires processing in the phago/lysosome and de novo class II MHC synthesis, whilst processing of another region of MSP1, for example within the less structurally constrained 38kd fragment can take place in recycling endosomes [7]. Here we examine the potential role of two proteinases, cathepsins D and E, in processing of the merozoite surface antigen, PcMSP1. These two enzymes are members of the aspartic acid proteinase family, and show considerable structural homology. However, they are located within different sub compartments of the endolysosomal system. Cathepsin D is a classical late endosome/lysosomal enzyme, targeted to this compartment via the mannose-6-phosphate receptor. In contrast, active cathepsin E is found predominantly in earlier endosomal structures [8]. In this study we combine genetic and pharmacological inhibition to probe the role of the two enzymes in processing of full length cell-associated PcMSP1 and soluble recombinant PcMSP1_21_ fragment. Unexpectedly, the context in which the protein is processed determines the role of these two enzymes in generating antigenic peptides leading to recognition by CD4 T cell hybridomas and IL-2 secretion.

## Results

**Figure 1:**
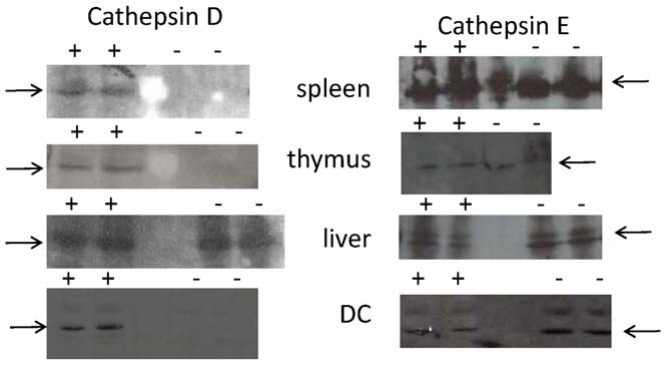
Haemopoeitic cells from CTSD −/− donor chimeras do not express CTSD. Spleen, thymus, liver and bone marrow DC from CTSD −/− and CTSD +/+ donor chimeras was fractionated by SDS-PAGE, and analysed by Western blot for expression of CTSD (left panels) and CTSE (right panels, as loading controls). One of four experiments.

Cathepsin D deficient mice die between days 20–23 of life due to progressive neuronal degeneration [9]. In order to study immunological function in healthy adult mice, we made radiation chimeras by transferring bone marrow cells from wild type or cathepsin D deficient BALB/c donors into lethally irradiated wild type BALB/c recipients. In these chimaeras the haematopoetic compartment is reconstituted by donor cells, and spleen, thymus and bone marrow-derived DC express undetectable levels of cathepsin D. In contrast, non- haematopoetic tissues such as liver show normal cathepsin D levels (Fig. 1). These mice are healthy, and do not develop any obvious neurological or other overt abnormality. The immune system of the mice contains normal numbers of T cells, B cells, DC and macrophages, have normal levels of antibody and normal T-dependent antibody response to ovalbumin and sheep RBC [10].

**Figure 2:**
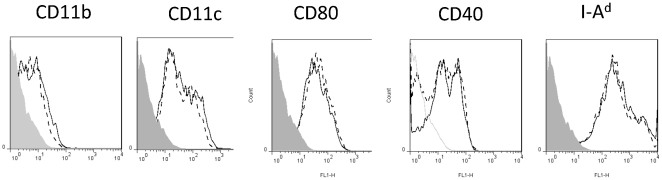
The phenotype of bone marrow derived DC is not affected by absence of CTSD. Bone marrow cells from CTSD −/− and CTSD +/+ donor chimeras were cultured in GM-CSF for seven days, purified on CD11c magnetic beads, and then analysed for expression of surface markers by flow cytometry. Cells were >80% CD11c positive. Shaded histogram: isotype control. Solid line : CTSD −/− DC. Dotted line CTSD +/+ DC. One of three experiments.

We first examined the ability of bone marrow generated DC from wildtype or cathepsin D-deficient chimaeras to process and present PcMSP1_21_ epitopes to specific CD4 T cell hybridomas, either from *P. chabaudi*-infected RBC, or from recombinant MSP1_21_ protein. Bone-marrow derived DC from deficient mice expressed no detectable cathepsin D, but were obtained in normal numbers, and had a similar cell surface phenotype to their cathepsin D expressing counterparts (Fig. 2).

The processing activity of the DC was assessed by measuring the response of the MSP1 specific T cell hybridomas, B7 and B5. The B7 epitope (MSP1_aa_ 1690–1709) is located within the C-terminal MSP1_21_ domain, while the B5 peptide (MSP1_aa_ 1157–1171) is located within the more N-terminal 38kd fragment.

**Figure 3:**
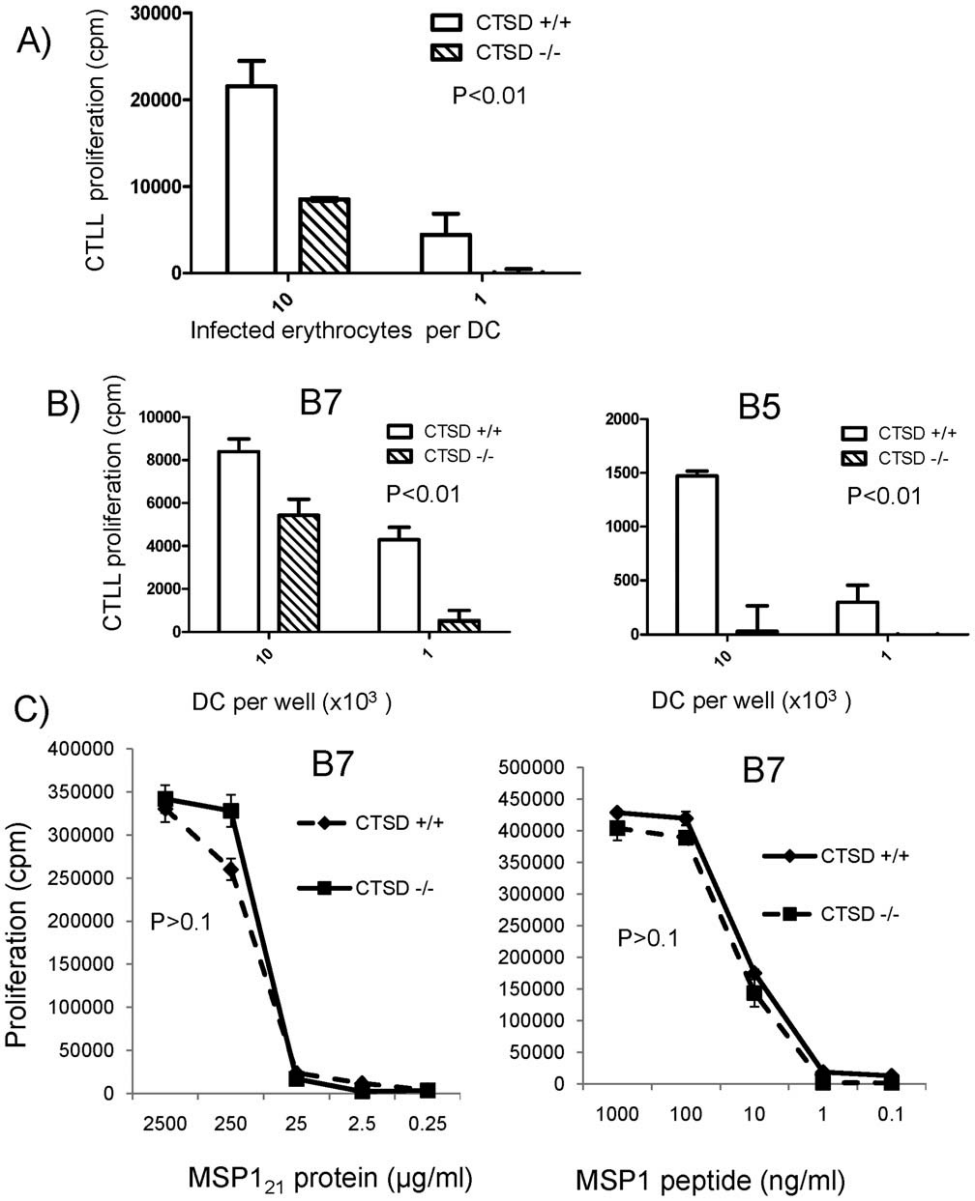
Absence of CTSD impairs the ability of DC to process erythrocytes infected with *P. chabaudi* merozoites in vitro. A) DC (10^3^) from CTSD −/− and CTSD +/+ donor chimeras were co-cultured with B7 T hybridoma cells (2×10^4^) and different numbers of iRBC. IL-2 release was measured after 24 hours using the indicator cell line CTLL. B) DC from CTSD −/− and CTSD +/+ donor chimeras were cocultured with iRBC (5 erythrocytes/DC) for three hours, and then different numbers of DC were co-cultured with B7 or B5 hybridoma cells as in A. C) DC (10^3^) from CTSD −/− and CTSD +/+ donor chimeras were co-cultured with B7 T hybridoma cells (2×10^4^) and different concentrations of MSP1 protein or a peptide coding for the B7 epitope at different concentrations. IL2 was release was measured as above. An additional intermediate concentration of MSP1 (50 µg/ml) was also tested in additional experiments with similar results. P values show the difference between CTSD +/+ and CTSD −/− analysed by Anova. One of four experiments.

Cathepsin D deficiency significantly impaired the ability of DC to process and present both the B7 epitope and B5 epitope of MSP1 from iRBC (Fig. 3A and 3B). In contrast, processing of recombinant protein MSP1_21_ or synthetic peptide was unaffected, and the IL-2 response of B7 was similar with both cathepsin D deficient and sufficient DC (Fig. 3C).

**Figure 4:**
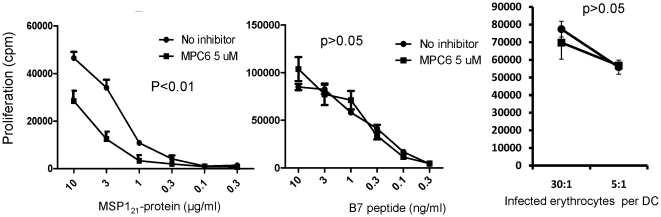
The aspartic proteinase inhibitor MPC6 inhibits processing of PcMSP1_21_. Bone marrow DC (10^3^) from CTSD +/+ donor chimeras were cocultured with differing concentrations of (A) PcMSP1_21_ protein or (B) a peptide coding the B7 epitope (1 µg/ml) and MPC6 (5 µM) for three hours and then colcultured with B7 hybridoma cells. p values show the significance value comparing presence and absence of inhibitor (Anova). One of three experiments. (C)_Bone marrow DC from CTSD +/+ donor chimeras were cocultured with iRBC (5 and 30 erythrocytes/DC) for three hours, and then the DC (10^3^) were cocultured with B7 hybridoma cells. p values show the significance value comparing presence and absence of inhibitor (Anova). One of two experiments.

Since we do not have access to a cathepsin E deficient mouse, we used a targeted inhibitor MPC6 [8], [11] to investigate whether the recombinant MSP1 protein was instead processed by cathepsin E. This inhibitor inhibits both cathepsin D and E, but no other known proteinase within DC. Inhibition of processing in cathepsin D deficient mice would therefore indicate a requirement for cathepsin E. MPC6 partially inhibited the processing/presentation of PcMSP1_21_, (Fig. 4), suggesting a requirement for cathepsin E in the processing of this protein. The inhibitor did not affect presentation of exogenously added B7 peptide, confirming that the effect was due to processing rather than to inhibition of some other later step in the antigen presenting pathway. We could not test the B5 hybridoma in this model, since the B5 epitope lies outside the PcMSP1_21_ fragment (cf [7]).

These data together suggest that the processing pathways/enzymes involved in processing of intact PcMSP1 on the surface of iRBC and the enzymes required to process the PcMSP1_21_recombinant protein fragment are different.

**Figure 5:**
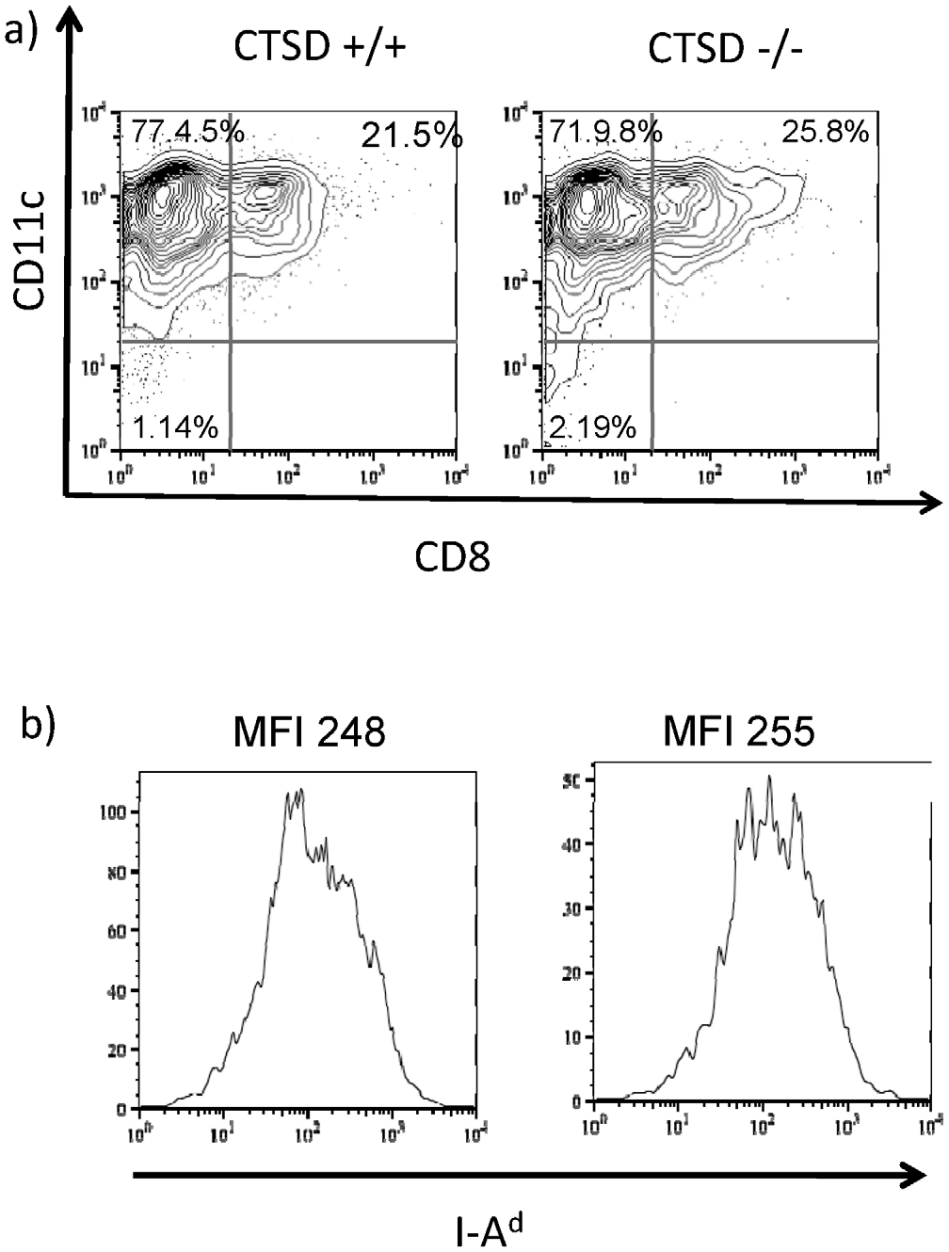
Normal numbers and class II MHC expression of DC isolated from spleens of *P. chabaudi* infected CTSD −/− and CTSD +/+ donor chimeras. DC were isolated from spleens of CTSD −/− and CTSD +/+ donor chimeras using magnetic CD11c beads, and stained for CD11c, CD8 and I-A^d^ using flow cytometry. Total numbers of cells isolated were similar in both sets of chimeras (3.83×10^6^ in CTSD +/+, and 4.5×10^6^ in CTSD −/− for experiment shown, one representative of two).

We next examined the effect of cathepsin D deficiency on processing of MSP1 *in vivo* in a *P. chabaudi* infection of BALB/c mice. Wildtype or cathepsin D deficient chimeras were infected with *P. chabaudi*. After 8 days, the mice were sacrificed and splenic CD11c+ DC were isolated by cell magnetic bead sorting (>90% CD11c purity) and cocultured with the B7 or B5 hybridomas. Proportion and numbers of DC subsets in the spleen were similar in both WT and cathepsin D in deficient mice at peak parasitemia (Fig. 5). In addition, class II levels on the DC were the same indicating that the DC recruitment and activation during malaria infection was not different in wt and cathepsin D deficient mice (Fig. 5).

**Figure 6:**
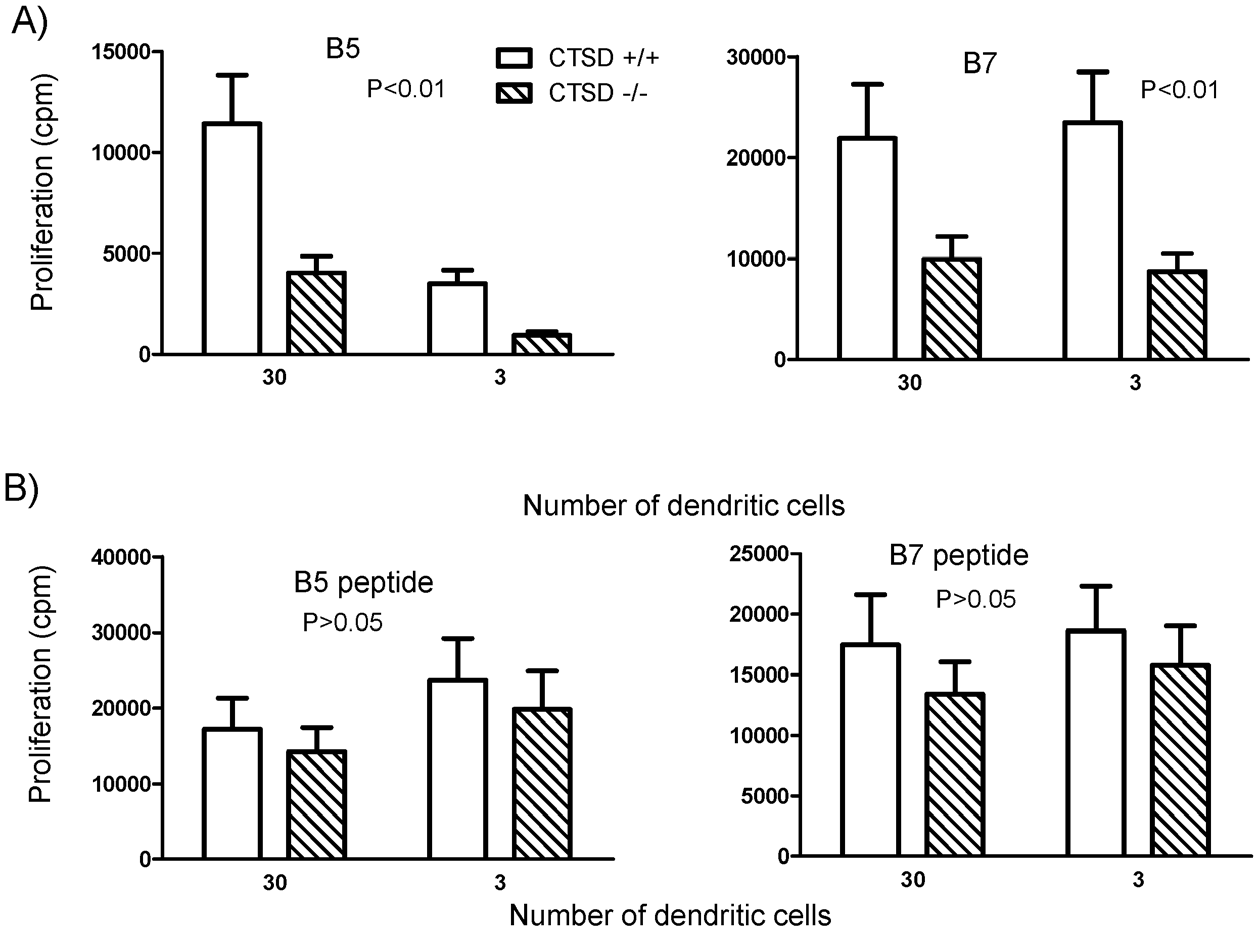
In vivo processing of MPS1 by CTSD −/− splenic DC is impaired. DC were isolated from spleens of CTSD −/− and CTSD +/+ donor chimeras 8 days after infection of the mice with *P. chabaudi*. A) The purified DC were co-cultured with B5 or B7 hybridoma cells and without any additional exogenous antigen and IL2 production measured after 24 hours, using the CTLL indicator cell line. B) As in A but peptide coding the B7 or B5 epitopes (1 µM) was added in vitro. P values for comparison of CTSD+/+ and CTSD −/− by ANOVA. One experiment of two.

**Figure 7:**
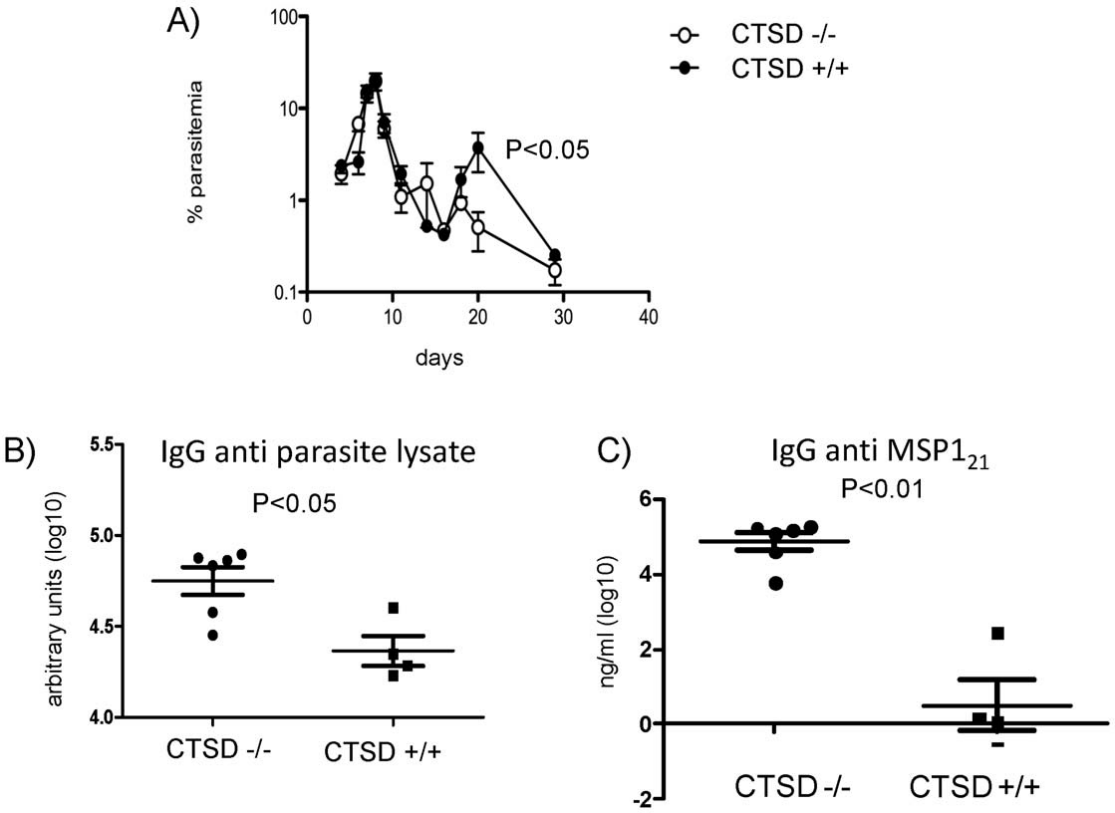
CTSD deficient, malaria infected mice have decreased late parasitaemia and enhanced malaria specific IgG responses. A) CTSD −/− and CTSD +/+ donor chimeras were infected with *P. chabaudi*, and percent parasitaemia measured at different days post infection as shown. Geometric mean and standard error of mean is shown (n = 6). Significance was measured using the Mann Whitney test. B) and C. As in A, but plasma was collected after 36 days for ELISA against whole parasite lysate (B) or PcMPS1_21_ protein (C). Each individual mouse, and the geometric mean and standard error of mean (n = 6) is shown. One experiment of two , each with six mice in each group.

DC from infected cathepsin D deficient mice were significantly less efficient at activating both B7 and B5 hybridomas (Fig. 6A). However, DC from both sets of mice were equally effective at activating the T cells when exogenous peptide antigen was added in vitro (Fig. 6B), indicating that the defect resulted from a reduced ability to process MSP1 antigen, and not a general defect in antigen presenting activity. Despite the reduced ability of DC from *P. chabaudi* - infected cathepsin D deficient mice in presenting MSP1 to T cell hybridomas, no significant differences were found in parasitemias between infected WT and cathepsin D deficient mice (Fig. 7A) in the first 16 days of infection. However later in infection, the cathepsin D deficient mice showed a reduced parasitemia.

IgG responses to whole parasite lysate and MSP1_21_ protein were measured in plasma from infected wildtype or cathepsin D deficient chimeric mice at day 36 post infection. Unexpectedly, antibody responses were enhanced in the absence of cathepsin D (Fig. 7B and 7C). This coincided with the reduced parasitemia, suggesting that the increased Ab response might be limiting parasitemia.

## Discussion

In this study we demonstrate that the absence of cathepsin D significantly inhibits the T cell response to two epitopes of *P. chabaudi cabaudi* MSP1 antigen, either after processing of infected erythrocytes by bone marrow derived DC *in vitro*, or by splenic DC during *in vivo* infection. By contrast, processing of soluble recombinant PcMPS1_21_ fragment is unaffected by the absence of cathepsin D, but is inhibited by an inhibitor which can inhibit both cathepsin D and E. This is the first reported example in which the processing mechanism for a specific epitope is shown to depend not just on its context within a protein, but on the context in which the protein is processed.

Cathepsin D is a lysosomal enzyme present at high concentrations within late endosomes, lysosomes and phago-lysosomes [12]. Its importance for lysosomal degradation is demonstrated by the fact that deficiency of the enzyme leads to a lysosomal-storage type disease, with progressive neural degeneration both in animals and man [13]. The role of cathepsin D in antigen processing has remained equivocal. Previous studies of antigen processing in cathepsin D deficient mice did not show any major defects [14], although one subsequent study suggested a role in processing of a Mycobacterial heat shock protein [15]. However, no previous studies have directly addressed a role for this enzyme in processing of particulate antigen, despite its known localisation to the phagolysosome. The data presented in this study are consistent with a model in which the malaria parasite, *P. chabaudi* in the form of free merozoites or within infected RBC are phagocytosed by DC (either *in vitro* or *in vivo*) and then processed to provide peptide epitopes for binding to class II MHC within the phagolysosome or endosome. Cathepsin D contributes to this processing step, although other enzymes can partially substitute for it, at least in its absence. In the case of the B7 epitope, which is within the disulfide bond-rich highly compact part of MSP1, processing may also require reduction of disulfide bonds, and the disulfide isomerise gamma-interferon-inducible lysosomal thiol reductase also found within late endosomes/lysosomes [16]. However, processing of both the B7 epitope within the compact PcMSP1_21_ domain, and B5, which is found within the less structured 38kd fragment, is reduced in the absence of cathepsin D. Thus this enzyme may play a dominant role in phagolysosomal protein fragmentation.

In contrast to cathepsin D, cathepsin E is absent from late endosomes/lysosomes, and is found either in immature form in the endoplasmic reticulum, or in a poorly defined sub-compartment of the early endosome [8]. The role of cathepsin E in antigen processing has been reported in several previous studies [8], [17]–[19], although a cathepsin E deficient mouse has only recently been generated [20]. Interestingly, the processing of different epitopes of ovalbumin show differential sensitivity to inhibition of cathepsin E, and the epitope which shows maximal cathepsin E requirement is found within a compact protein domain which is tightly folded with disulfide bonds similar to the B7 epitope within MSP1. Further studies will be required to determine whether this is a general feature of cathepsin E sensitive epitopes.

A paradoxical finding of our study was that the absence of cathepsin D leads to reduced processing, but does not impair either malaria specific IgG production or parasite clearance. Indeed the IgG levels against MSP1_21_ and whole parasite lysate appear enhanced, and parasite numbers at later times points slightly reduced. The mechanisms underlying this observation must remain speculative. However, absence of cathepsin D is likely to have broader effects on both self and non-self repertoire of the CD4 compartment than just the two epitopes investigated in this study. This in turn may affect both the qualitative and quantitative nature of the overall T cell response to the *P.chabaudi* infection. Further studies will be required to understand this effect fully. The observation highlights our limited understanding of the mechanisms which underly antigen processing, how this determines the repertoire of the responding T cells, and how in turn this is reflected in host immunity. Increased understanding of all these steps is important for designing more effective immunological strategies to control infection by malaria.

## Materials and Methods

*Plasmodium chabaudi chabaudi* clone AS (originally obtained from Dr. K.N. Brown, National Institute for Medical Research, London) was routinely injected from frozen stocks. Infections were initiated by intraperitoenal injection of 10^5^ RBC (infected red blood cells) obtained from infected mice before the peak of parasitaemia, as described previously [21]. Thin blood films were made from tail blood and stained with Giemsa R66 (BDH, Poole, GB) solution to monitor the course of parasitaemia.

Isolation of *P. chabaudi infected* RBC (iRBC). Blood was collected from BALB/c mice infected with *P. chabaudi* at day 7 of infection. Contaminating leukocytes were removed using Plasmodipur filters (Euro-Diagnostica, UK) and late trophozoite stage iRBC were enriched by centrifugation on 74% isotonic Percoll (Amersham Biosciences,UK) as described previously [22]. Purity of trophozoite-infected RBC as assessed by Giemsa was greater than 95% pure.

Recombinant PcMSP121 protein was expressed in *Pichia pastoris* as described previously [5].

Mice. BALB/c mice (BALB/c Ola Hsd) were purchased from Harlan UK and kept in the Biological Services, UCL. Mice carrying a neomycin insertion in the cathepsin D gene [9] were bred onto the BALB/c background for ten generations, and then maintained as a heterozygote inbred colony. Homozygotes could be identified by day 19 or 20 by their small size, and reduced mobility. Genotype was confirmed in each case by PCR as described. All experiments were carried out under UK Animal Project Licence authorization.

Bone marrow radiation chimaeras. Recipient BALB/c mice were kept on acidified water (0.01% conc. HCl in H_2_0) for 1 week prior to transfer. The mice were irradiated with 8 Gy (delivered over 15–30 minutes) with an X-ray source (A.G.O. HS X-RAY SYSTEM, Reading, UK) and then allowed to recover for 4–5 hours, before receiving 2×10^6^ bone marrow cells from cathepsin D deficient, or wild type litter-mates, intravenously in 0.2 ml PBS. The chimaeric mice were maintained for a minimum of 2–3 months in order to allow full reconstitution of the immune system [10].

Dendritic cell culture. Dendritic cells were obtained by culture of bone marrow cells (5×10^5^/ml) in Iscove's medium (Gibco BRL (Invitrogen Life Technologies/Life Sciences), Paisley, UK), 10% FCS (Gibco BRL) with the addition of GM-CSF (20 ng/ml; PeproTech, Rocky Hill, NJ, USA). Fresh medium and cytokine were added on day 4 and dendritic cells were harvested on days 7/8. Dendritic cells were further purified by magnetic cell sorting, using mouse CD11c^+^ microbeads (CD11c (N418), Miltenyi Biotec, Bergisch Gladbach, Germany) and the appropriate columns (MS separation columns, Miltenyi Biotec), according to the manufacture's guidelines. The enriched population was 85 to 95% CD11c^+^.

Measurement of P. chabaudi and PcMSP1**_21_** specific IgG antibodies. Plasma was collected 36 days after a primary *P. chabaudi* infection. ELISA assays to measure malaria-specific IgG antibodies were performed as described previously [5], [6]. For the MSP1 ELISA, monoclonal anti MSP1_21_ antibody, NIMP23 was used as standard for determining the concentration of MSP1_21_-specific IgG [23] . *Plasmodium chabaudi* hyperimmune plasma was used as the standard to determine anti parasite lysate IgG and its antibody binding capacity was defined as 1000 U [6]. Plasma from uninfected BALB/c mice was used as a negative control. Specific IgG antibodies were detected using alkaline phosphatase (AP)-conjugated goat anti-mouse IgG antibodies (Southern Biotechnology, Birmingham, AL, USA) and *p*-nitrophenyl phosphate substrate (PNPP, Sigma). The data are represented as the mean IgG antibody units from individual mice.

Splenic dendritic cells. Spleens were dissected from mice at day 8 after i.p. infection with 10^5^ *P. chabaudi*, and treated for 30 min at 37°C with 0.4 mg/ml of Liberase CI (Roche, Basel, Switzerland) in serum-free Iscove's Modified Dulbecco Medium (IMDM) (Sigma, Dorset, Poole,UK). After washing and centrifugation at 500 g for 10 min, cell pellets were re-suspended for 5 min in 8.3 g/L NH_4_Cl in 0.01 M Tris-HCl pH 7.5 to lyse RBC. Spleen cells were washed, incubated with Fc receptor (FcR) block followed by anti-CD11c magnetic beads and enrichment on magnetic columns (Miltenyi Biotec, Bergisch Gladbach, Germany) to more than 95% purity.

Western blotting. Western blots were performed on liver, spleen thymus or BMDC homogenate, prepared using a Dounce homoginiser. Protein (30 ug as measured by Bradford assay) were loaded in each lane and separated by 12% denaturing SDS polyacrylamide gel electrophoresis (PAGE) under reducing conditions. Proteins were transferred electrophoretically to nitrocellulose membranes and then immunostained using standard procedures. Primary antibodies used were: monoclonal rat anti-mouse Cathepsin D (R&D System, Minneapolis, MN, US, 4 µg/ml); rabbit anti-rat Cathepsin E antibody (WAKO, Neuss, Germany), diluted 1∶1000. Primary antibodies were detected using HRP-conjugated rabbit anti-rat IgG or goat anti-rabbit IgG (DAKO, Glostrup, Denmark, diluted 1∶2000), and ECL detection reagent (Amersham Pharmacia Biotech, Bucks, UK). For quantification, gels were scanned and digital images analyzed using Image J software [24].

Cell lines , peptides and MPC6. CTLL-2 cells, B5 hybridoma recognising aa 1137–1151 in the p38 fragment of MSP1 and B7 T cell hybridoma recognising aa 1673–1694 in the MSP1_21_ fragment [7] were cultured in IMDM (Sigma, Dorset, Poole) supplemented with 10 percent FCS, 1 mM L-glutamine, 10 mM Hepes, 5×10^−5^ M 2-Mercaptoethanol, 100 µg/ml penicillin,100 U/ml streptomycin and 1 mM sodium pyruvate. CTLL-2 cells were cultured with 10 U/ml human recombinant IL-2. B5 peptide (ISVLKSRLLKRKKYI) and the B7 peptide (RCEKDTEATCSINKGGCDPS) were synthesized by Jerini AG. Berlin. MPC6 was synthesised as previously described [11].

Flow cytometry Single cell suspensions of purified CD11c+ DC or bone marrow derived DC were resuspended in FACS buffer (0.5% w/v BSA, 2 mM EDTA, 0.05% sodium azide in PBS). 5×10^5^ cells were pre-incubated at room temp for 10 min with anti-Fc R block. All other antibody incubation steps were 20 min on ice. The following antibodies were used, at concentrations recommended by manufacturer : CD11b APC conjugate, clone M1/70, BD pharm, isotype: rat IgG2b; CD11c: PE-Cy7 conjugate, clone N418, eBioscience, isotype: hamster IgG ; 3. CD40 FITC conjugate, clone HM40-3, BD pharm, isotype: IgM; CD80:

FITC conjugate, clone 16-10A1, BD pharm, isotype: hamster IgG2 I-Ad.

The cells were acquired on a BD LSR II Flow Cytometer (BD Biosciences) and analysed with FlowJo software (TriStar).

Antigen presentation assay using B5 and B7 T cell hybridomas. Different numbers of bone marrow or splenic CD11c+ DCs were incubated with 2×10^4^ T cell hybridoma cells (Costar), in a 7 percent CO2 incubator and at 37°C for 24 h. As positive controls, similar numbers of DCs were incubated with 1 µM peptide. The supernatants were then collected, frozen, thawed and added to 5000 CTLL-2 cells per well in IMDM supplemented with 10 percent FCS in 96 well plates. After 24 h incubation, the cells were pulsed with 1 µCi ^3^H Thymidine (Amersham) for 16 hr and proliferation determined as previously described [6].
